# Necrotizing fasciitis following saphenofemoral junction ligation with long saphenous vein stripping: a case report

**DOI:** 10.1186/1752-1947-4-161

**Published:** 2010-05-27

**Authors:** Stella Ruth Smith, Moayad Aljarabah, Graeme Ferguson, Zahir Babar

**Affiliations:** 1 Department of Surgery and Anaesthesia, Royal Bolton NHS Foundation Trust, Minerva Road, Farnworth, Bolton, Lancashire BL4 0JR, UK

## Abstract

**Introduction:**

Necrotizing fasciitis is a rare condition with a mortality rate of around 34%. It can be mono- or polymicrobial in origin. Monomicrobial infections are usually due to group A streptococcus and their incidence is on the rise. They normally occur in healthy individuals with a history of trauma, surgery or intravenous drug use. Post-operative necrotizing fasciitis is rare but accounts for 9 to 28% of all necrotizing fasciitis. The incidence of wound infection following saphenofemoral junction ligation and vein stripping is said to be less than 3%, although this complication is probably under-reported. We describe a case of group A streptococcus necrotizing fasciitis following saphenofemoral junction ligation and vein stripping.

**Case Presentation:**

A 39-year-old woman presented three days following a left sided saphenofemoral junction ligation with long saphenous vein stripping at another institution. She had a three day history of fever, rigors and swelling of the left leg. She was pyrexial and shocked. She had a very tender, swollen left groin and thigh, with a small blister anteriorly and was in acute renal failure. She was prescribed intravenous penicillin and diagnosed with necrotizing fasciitis. She underwent extensive debridement of her left thigh and was commenced on clindamycin and imipenem. Post-operatively, she required ventilatory and inotropic support with continuous veno-venous haemofiltration. An examination 12 hours after surgery showed no requirement for further debridement. A group A streptococcus, sensitive to penicillin, was isolated from the debrided tissue. A vacuum assisted closure device was fitted to the clean thigh wound on day four and split-skin-grafting was performed on day eight. On day 13, a wound inspection revealed that more than 90% of the graft had taken. Antibiotics were stopped on day 20 and she was discharged on day 22.

**Conclusion:**

Necrotizing fasciitis is a very serious complication for a relatively minor, elective procedure. To the best of our knowledge, this is the first report in the English-language literature of this complication arising from a standard saphenofemoral junction ligation and vein stripping. It highlights the need to be circumspect when offering patients surgery for non-life-threatening conditions.

## Introduction

Necrotizing fasciitis is a rare condition that is characterised by widespread necrosis of fascia and subcutaneous tissue. The incidence is around three cases per 10,000 hospital admissions in the USA [1]. The mortality rate is high, around 34% (range 6-76%) [1-4]. Swift diagnosis is extremely important as the primary determinant of mortality is time to operative intervention [4,5]. Other variables associated with mortality are shown in table 1. Additionally, necrotizing fasciitis often results in serious complications such as adult-respiratory distress syndrome (ARDS), acute renal failure, cardiac failure and concomitant nosocomial infections.

**Table 1:** Factors associated with increased mortality in necrotizing fasciitis [1-5,8].

Patient factors	Other factors
Age > 60 years	Time to operative intervention (surgery delayed >24 hours correlates with relative risk = 9.4 (p < 0.05)) [8]

Female gender	Inadequacy of initial debridement

Intravenous drug use	Larger percentage of body surface involved

Diabetes with peripheral vascular disease or chronic renal failure	Multi-organ dysfunction - the more organs failed on admission, the worse the prognosis

Other co-morbidities, particularly cancer, congestive cardiac failure, peripheral vascular disease, intravenous drug abuse, pulmonary disease	Shock, coagulopathy or acidosis on admission

	WCC >30 cells/mm^3 ^on admission

	Acute renal failure on admission (doubles the mortality risk) [4]

	Clostridial or vibrio vulnificus infection

Necrotizing fasciitis is often categorized according to the microbial source. Type I infections are polymicrobial and tend to occur in patients with significant co-morbidities. Type II infections are caused by group A streptococcus and are most common in otherwise healthy individuals with a history of trauma, intravenous drug abuse or surgery [5]. Post-operative necrotizing fasciitis is rare but accounts for 9-28% of all necrotizing fasciitis [1,3,4]. Wound infections following traditional saphenofemoral junction ligation and vein stripping are said to occur in less than 3% of cases and necrotizing fasciitis following this operation has not been described in the English-language literature [6,7]. We describe a case of type II necrotizing fasciitis following saphenofemoral junction ligation and vein stripping.

### Case Presentation

**Figure 1:**
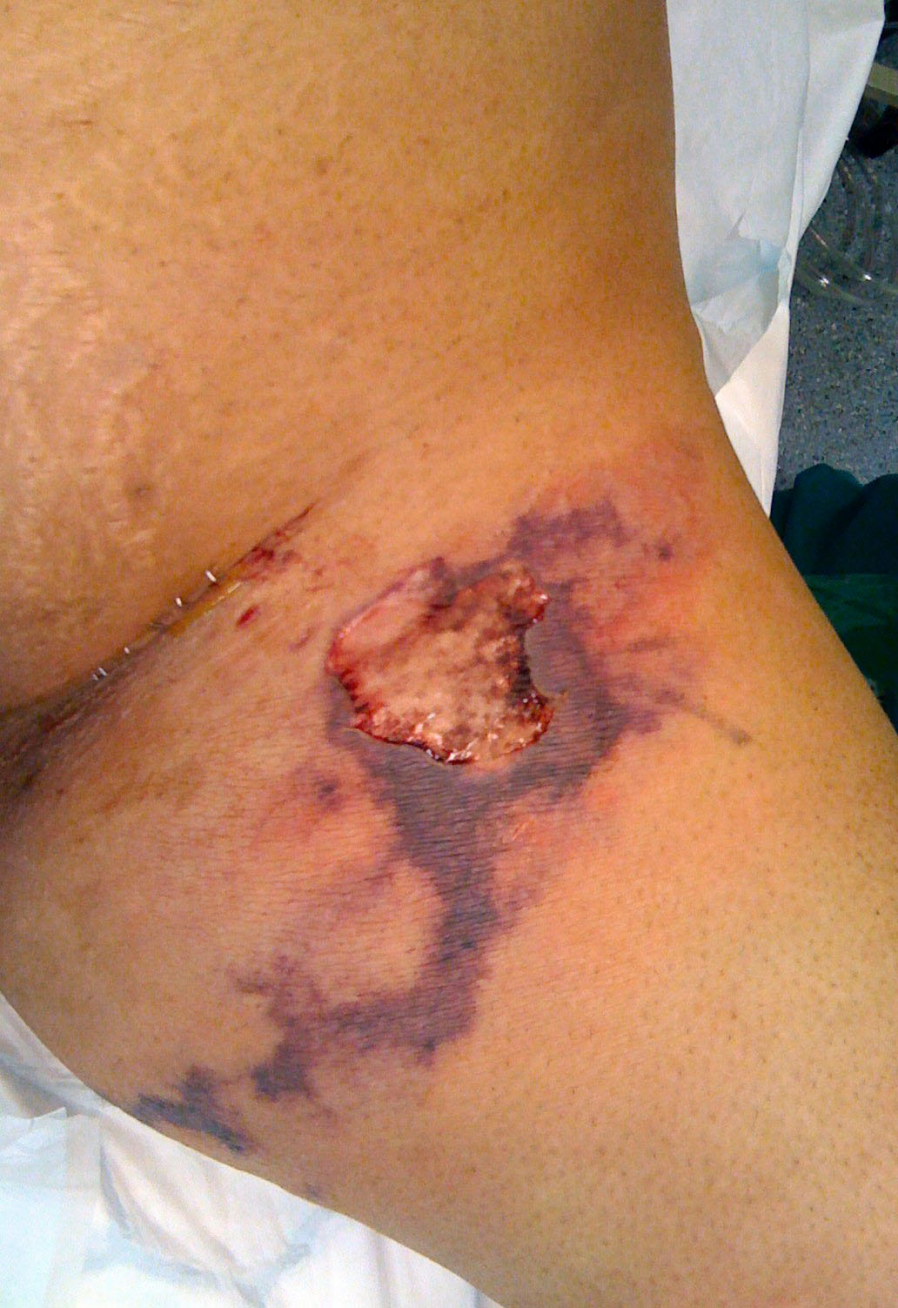
**Pre-operative appearance of necrotizing fasciitis of the left groin and thigh three days following saphenofemoral junction ligation and long saphenous vein stripping**.

A 39-year-old woman, with no co-morbidity, underwent a left-sided saphenofemoral junction ligation, long saphenous vein stripping and multiple avulsions at another institution for recurrent thrombophlebitis. She presented three days post-operatively with a three day history of fever, rigors and swelling of the left leg. On examination she was noted to be pyrexial at 38.0°C, tachycardic and hypotensive. She had a very tender, swollen left groin and thigh with a blister anteriorly. Her white cell count was initially normal (8.4 × 10^9^/L). She was in acute renal failure (urea 17.7 mmol/L, creatinine 326 mmol/L) with a metabolic acidosis (pH 7.30, lactate 5.9, base excess -10.3, bicarbonate 16.9). Amoxicillin 1 g with flucloxacillin 1 g was administered intravenously. Further examination revealed progressive skin necrosis, see figure 1. A diagnosis of necrotizing fasciitis was made and she was taken to theatre.

**Figure 2:**
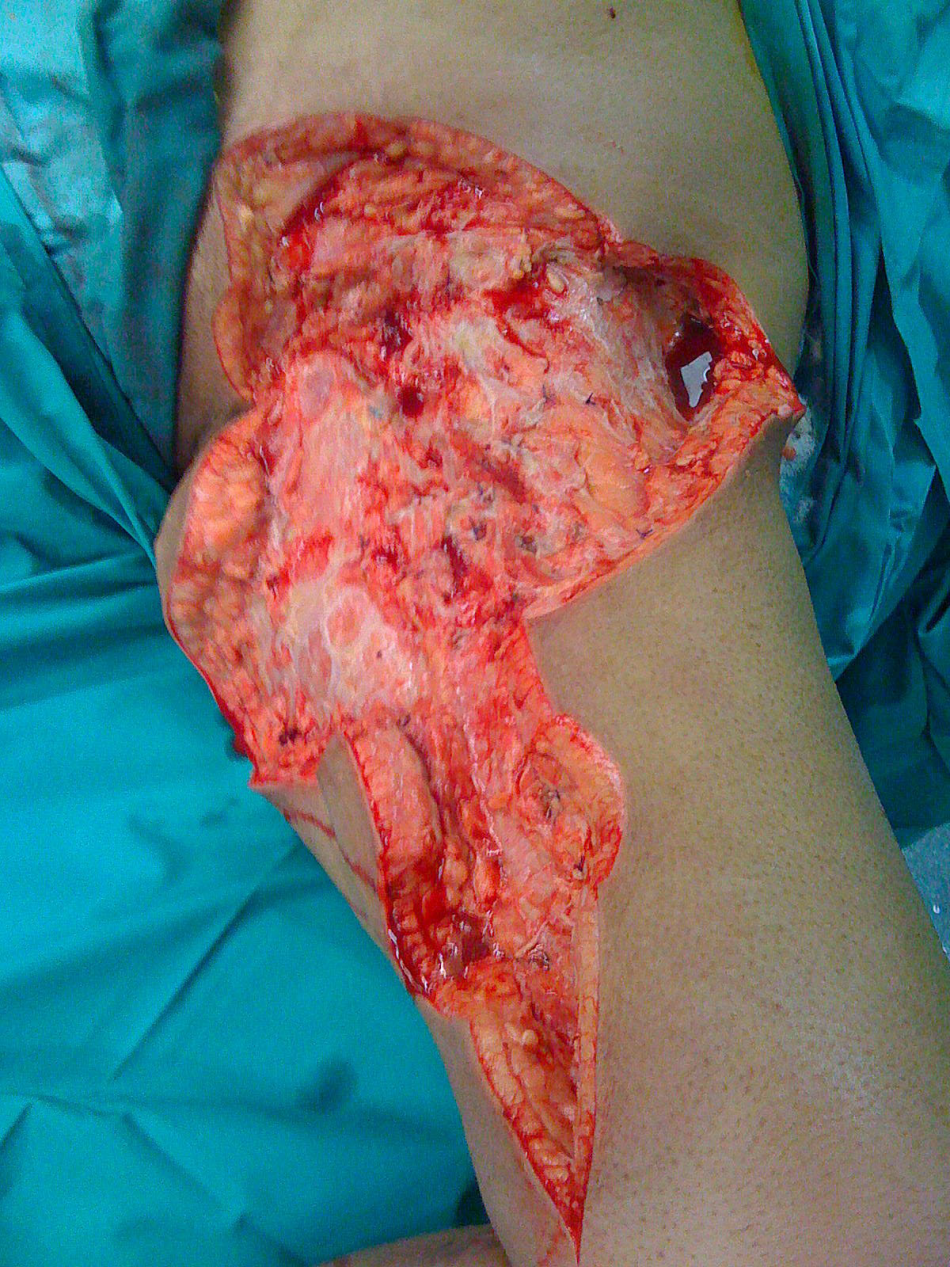
**Extent of initial debridement of left anteromedial thigh**.

At operation her groin wound was explored and pus was noted to be tracking down the site of the stripped vein. A large area of necrotic tissue, including necrotic fascia, was debrided, see figure 2. Tissue was sent for urgent microscopy, culture and sensitivity. Clindamycin 900 mg tds and imipenem 1 g tds was commenced. Post-operatively, she required ventilatory and inotropic support. She was coagulopathic (international normalised ratio 2.0) and required four units of fresh frozen plasma. Her white cell count dropped to 1.4 × 10^9^/L but normalised the following day. Overnight her acidosis worsened (pH 7.19), despite a good urine output. Sodium bicarbonate was given and continuous veno-venous haemofiltration begun. The wound was examined 12 hours post-surgery on the intensive care unit. The wound edges were healthy with bleeding and, therefore, no further debridement was undertaken.

She was extubated on day two and did not require further haemofiltration. Inotropic support continued for three days. A wound review in theatre on day two revealed a clean wound and plans were made for skin grafting the following week. A group A streptococcus was isolated from the debrided tissue. It was sensitive to penicillin, so imipenem was converted to benzylpenicillin 1.2 g qds on day three. Only one set of blood cultures were taken on admission and no growth was isolated from them. A vacuum assisted closure device was fitted to the clean thigh wound on day four.

**Figure 3:**
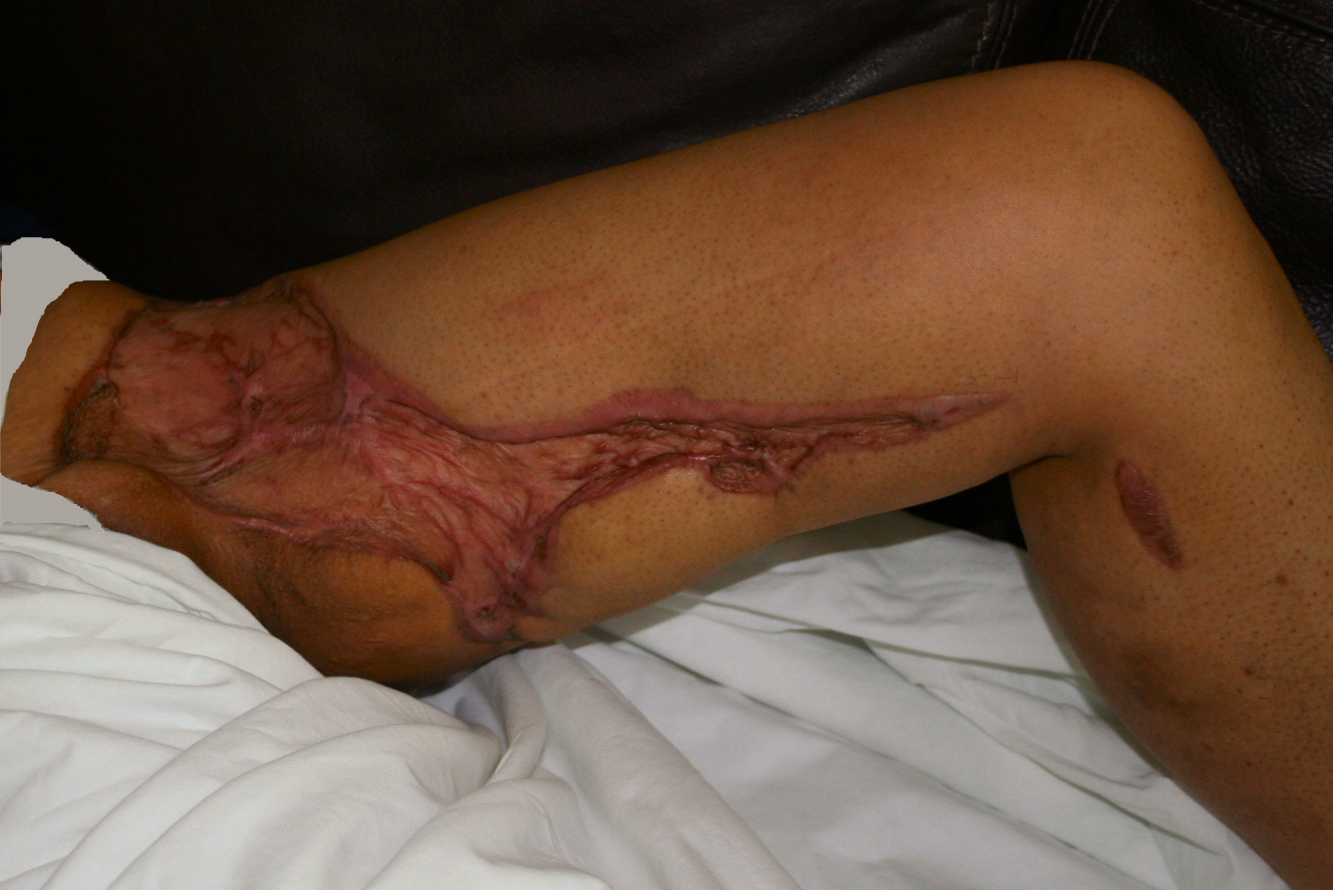
**Left anteromedial thigh two months following skin grafting**.

Split-skin-grafting was performed on day eight using the right thigh as a donor site. On day 13 her wound was inspected, revealing that >90% of the graft had taken and there was no clinically apparent infection. Oral antibiotic therapy with clindamycin 300 mg tds and amoxicillin 500 mg tds was begun. All antibiotics were stopped on day 20 and she was discharged on day 22. Significant scaring has resulted, see figure 3. It has taken three months for her to get back to work full-time and for her mobility to return to normal.

## Discussion

Necrotizing fasciitis can be classified according to the anatomy involved, the depth of infection or the microbial source, although these systems do not affect diagnosis or management [5]. The majority of necrotizing fasciitis (around 70%) is polymicrobial in nature (type I), with an average of three to four different organisms [1,3,8]. These organisms are usually a combination of gram-positive cocci (usually *Staphyloccous aureus *or *Streptococcus*), gram-negative rods and anaerobes [2,3,5,9]. These infections are more common in patients with co-morbidities such as diabetes, peripheral vascular disease, intravenous drug abuse, immunocompromise (HIV, steroids) and chronic renal failure [5,8]. Other risk factors include obesity, trauma (blunt or penetrating injury, insect bites, surgical wounds) and perforation of the gastrointestinal tract [1,5]. However, in 15-50% patients, no specific cause can be found [1-3,5].

Type II necrotizing fasciitis is monomicrobial and much less common. It usually occurs in otherwise healthy individuals and is often associated with trauma, surgery or intravenous drug abuse [3]. The most common causative organism is group A streptococcus, but staphylococcus aureus may be isolated concurrently. Despite its historical significance, *Clostridium perfringens *is now a rare cause of necrotizing fasciitis due to improvements in sanitation and hygiene. Our patient grew *Streptococcus pyogenes *from her wound. She was typical of a patient with type II infection as she was fit and healthy with a recent history of surgery.

The incidence of invasive group A streptococcal infections (including necrotizing fasciitis, septic arthritis, meningitis) is increasing, although it is not clear why [3,10]. There is a normal seasonal variation, with infection most common in December to April. However, intermittent upsurges have occurred; one in 2003 was associated with intravenous drug use [10]. The winter of 2008 to 2009 has also shown an increase in infection rates, but no attributing cause has yet been found [10]. However, the mortality rate from necrotizing streptococcal infection is not significantly different from fasciitis from other causes [3].

The association between necrotizing fasciitis and surgery is established, but rare. It is the causative factor in 9%-28% of all types of necrotizing fasciitis and usually occurs with significant faecal contamination [1,3,4]. For traditional saphenofemoral junction ligation, long saphenous vein stripping and avulsions, the incidence of wound infection is said to be less than 3% [6,7]. However, these infections may be under-reported as they will often be treated in the community. One report suggests that the infection rate may be as high as 14% [11]. There is only one report of necrotizing fasciitis following long saphenous vein stripping and this was performed under tumescent anaesthesia (large-volume infiltration of a combination of lidocaine, epinephrine, triamcinolone, sodium bicarbonate and normal saline) [12]. In this case, the patient had diabetes, hypertension, severe coronary artery disease and hyperuricaemia which the authors suggest should contraindicate the use of tumescent anaesthesia [12]. We could find no reports in the English-language literature of necrotizing infection following this operation under general anaesthetic.

Most commonly, patients present with erythema and swelling with exquisite pain and tenderness that extends beyond the erythema. The pain is usually disproportionate to the physical signs. Later signs include bullae, induration, fluctuance, crepitus, necrosis or sensory deficit and are indicative of a worse prognosis. Patients are usually systemically unwell with a pyrexia and can rapidly progress to multi-organ failure. Unfortunately, a large proportion of patients are initially misdiagnosed with a simple soft-tissue infection (86% of cases in a retrospective study of 89 patients) [8]. The paucity of early signs (there may not be any cellulitis) and the rarity of the disease contributes to this problem. Our patient had a normal white cell count on admission which dropped to very low levels following her debridement. This demonstrates the point that overwhelming sepsis can result in a normal or low white cell count.

Where the diagnosis is in question, a "finger test" can be performed. This involves infiltrating with local anaesthetic and making a 2 cm incision down to deep fascia. Lack of bleeding and "murky dishwater" fluid are ominous signs. A positive finger test is defined by lack of resistance of the subcutaneous tissues to finger dissection off the deep fascia. Rapid frozen section tissue biopsies can be sent, but most practitioners advocate a rapid debridement. Radiological studies are occasionally used but should not be performed routinely as this delays treatment. The gold-standard diagnostic modality is operative exploration. As with the finger-test, positive findings include "murky dishwater" fluid, grey, necrotic fascia, lack of bleeding and loss of the normal resistance of the fascia to dissection. Intra-operative tissue biopsy with gram-stain or a frozen-section can be performed, but is not usually necessary as the diagnosis is clear.

Successful treatment of necrotizing fasciitis involves removing all the necrotic tissue, beginning broad spectrum antibiotics immediately and supporting failing organs. The most important determinant of mortality is the timing and adequacy of initial debridement [1,8]. The skin edges should be free from cellulitis, healthy and bleeding. The amount of debridement is often much larger than is appreciated on physical examination as the infection tracks along the fascia. Serial debridements are usually required, spaced 12-36 hours apart.

Amputation is needed in up to 26% of extremity necrotizing fasciitis, either to gain control of ascending infection or to remove a functionless limb when large volumes of muscle have been debrided [1-3,5]. Perianal, perineal and scrotal infections can benefit from a temporary diverting colostomy to reduce the number of dressing changes, prevent superimposed infection and protect skin grafts.

Wounds are usually left open and treated with simple dressings initially. There is little evidence for the use of iodine solutions or antibiotic solutions [5]. Vacuum-assisted closure devices have become popular. They are thought to enhance granulation tissue formation, reduce the surface area of the wound and reduce the time to healing up to four-fold, although there are no large studies evaluating their role in necrotizing fasciitis [5,13]. Skin-grafting is commonly required once the wound is clean and granulated. Sometimes reconstruction with full-thickness, free or rotational flaps is needed.

Antibiotics reduce systemic sepsis and bacterial spread and should therefore be started immediately, but they do not penetrate the infected, necrotic tissue due to the thrombogenic nature of the disease process. Initial antibiotic regimens should be as broad-spectrum as possible as the majority of necrotizing fasciitis is polymicrobial. Traditionally, regimens of choice included high-dose penicillin with clindamycin, as was used in our patient. This regimen covers gram-positive and anaerobic organisms, and, specifically, clostridia. Additional cover of gram-negative organisms could be provided with a third agent such as an aminoglycoside. Monotherapy with agents such as imipenem, tigecycline™ or tazocin™ has been described [9]. Concerns regarding resistance, particularly methicillin-resistant staphylococcus aureus (MRSA), have led to the introduction of drugs such as vancomycin and linezolid as first-line empirical cover for gram-positive organisms.

Immune globulin therapy (mainly immunoglobulin G) has been tried in some small studies [9]. It theoretically binds staphylococcal- and streptococcal-derived exotoxin, limiting the systemic cytokine response. It is costly and it not currently licensed in the UK or USA for this use. The role of hyperbaric oxygen therapy is unproven. It may be of benefit in patients with clostridial infections where increased oxygen tension results in decreased exotoxin elaboration [5].

## Conclusion

Necrotizing fasciitis is a very serious complication for a relatively minor, elective procedure. Our patient had a good indication for surgery: recurrent thrombophlebitis. However, she has suffered physically and emotionally from her disastrous complication. She has had to take a number of months off work and is left with significant scarring. This case serves to highlight the need to be circumspect when offering patients surgery for non-life-threatening conditions.

### Consent

Written informed consent was obtained from the patient for publication of this case report and accompanying images. A copy of the written consent is available for review by the Editor-in-Chief of this journal.

## Competing interests

The authors declare that they have no competing interests. No financial support has been received to help prepare this manuscript.

## Authors' contributions

SS conceived the report, collected data and drafted the manuscript. All authors critically appraised the manuscript and approved the final text.

## About Authors

Ms Stella Smith is a Specialist Registrar in General Surgery. Mr Moayad Aljarabah is a Registrar in General Surgery. Mr Graeme Ferguson is a Consultant Vascular Surgeon at the Royal Bolton NHS Foundation Trust. Mr Zahir Babar is an Associate Specialist in Plastic Surgery.
